# United States' Infrastructure Bill Contains Hidden $15 Billion Investment in Violence Prevention: Lead Abatement

**DOI:** 10.3389/fpubh.2022.885460

**Published:** 2022-07-07

**Authors:** Kyle R. Fischer, Erin Walton, Gregory N. Jasani

**Affiliations:** ^1^Department of Emergency Medicine, University of Maryland School of Medicine, Baltimore, MD, United States; ^2^Department of Trauma, R Adams Cowley Shock Trauma Center, Baltimore, MD, United States

**Keywords:** violence prevention, lead, gun violence, health policy, public health, environmental health

Recently, the United States' Congress passed and President Joseph Biden signed a long-anticipated, bipartisan, “Infrastructure Investment and Jobs Act” (1). The legislation includes ~$1 trillion for infrastructure updates and modernization. Although many have focused on its funding for transportation projects, namely roads and bridges, there is another rarely discussed benefit the bill is likely to provide: reducing community violence. That is because the final bill included $15 billion dedicated to lead pipe removal.

Lead is a naturally occurring heavy metal that until recent decades was commonly utilized in gasoline, household paint, and water service lines. We now know that lead is a poisonous metal and a devastating neurotoxin. This knowledge led to governmental actions that forbids lead content in all three sources (2). In the population of gunshot survivors, retained bullet fragments act as a source of direct toxicity as well (3). Despite this understanding, the United States still maintains a water supply that utilizes 6.1 million lead service lines today (4). The result is an ongoing public health problem.

It may seem odd to think of lead pipes and violent crime as linked, but evidence indicates that exposure to lead in childhood is a risk factor for violence later in life (5, 6). This should not be surprising as the effects of lead on brain development are well documented in the scientific literature. Specifically, lead affects the development of children's cerebellum, hippocampus, and prefrontal cortex. These are areas of the brain that are critical for cognition, behavior and memory.

Although causes of violence are multifactorial, including issues as entrenched as poverty, racial inequity, and disparities in policing and education, violent crime is often an impulsive act. Given the neurocognitive effects of lead exposure, it is no surprise that research has shown an association between lead exposure and criminal justice system involvement (7). Robust data from the economic literature provides convincing evidence of the causal effects of the toxins on risky, antisocial behaviors, such as violence (5). Taken together, many experts now consider prior lead abatement measures to be a significant component of the “Great American Crime Decline” of the 1990s (8).

To be clear, combatting community violence in our communities will require a “health in all policies” approach to address the deeply entrenched racial inequities. While childhood lead exposure is a notable risk factor for violence, it is not the primary driver. Decades of policy, rooted in structural racism, have progressively compounded the challenge. For example, research examining the effect of racial discrimination in the 1930s, commonly known as redlining, show persistent adverse effects on the social determinates of health, including elevated levels of gun violence nearly a century later (9).

A truly effective response to community violence will require a multipronged investment in these same communities to reverse the innumerable policy decisions and chronic disinvestment. These strategies range from traditional public health programming such as deploying violence intervention and prevention programs (10) to altering health system priorities through insurance policy (11). From an environmental health lens, the work of Dr. Eugenia South is instructive. By deploying place-based interventions, such as structural home repairs, to neighborhoods experiencing segregation and concentrated poverty, these targeted investments translated into decreases in assault and homicide (12).

Although only a portion of a comprehensive solution, environmental interventions represent an achievable component of violence prevention strategy. Specifically, lead abatement remains a significant opportunity for tangible public health benefits. Although prior measures, such as removal from gasoline provided broad public health benefits, remaining sources of exposure exacerbate significant health disparities. To date, Black children hold four-fold higher odds of being found to have an elevated blood lead level greater than U.S. Centers for Disease Control and Prevention recommendations compared to their White or Hispanic peers (13). With regards to lead service lines, the disparities appear to also occur geographically, with the U.S. industrial Midwest disproportionately utilizing remaining lead service lines (4).

The Infrastructure bill's funding for lead removal will allow municipalities the opportunity to provide targeted investments in previously underserved communities, especially communities of color. This allows for a strategic investment in both long-term health and safety.

Policymakers would be wise to consider unintended consequences as they distribute this funding. Baltimore City, which has captured escalating national attention for its rates of violent crime, serves as a case example. Due to the number of people living in poverty in Baltimore City, along with the aging stock of public, subsidized, and affordable housing, over 65,000 children were diagnosed with clinically significant blood-lead levels of serious concern between 1993 and 2013 (14). A large segment of this exposure was related to research projects that were conducted in the 1990s in which families with children were moved into homes with known lead levels in order to test abatement processes (15).

Later, lawsuits filed by those families claimed that they were targeted for participation because they were low-income, and that they were not made aware of the risks of exposure to lead prior to enrolling in the experiment (16). Additionally, landlords continued to knowingly and unlawfully sell and rent unabated homes to families who could not afford to negotiate or move. This resulted in financial settlements of ~$300 million. One Baltimore lawyer, who handled more than 4,000 lead poisoning lawsuits over 30 years, stated that 99.9% of his clients were African American (17).

Clients who received settlements were awarded structured payments up to thousands of dollars per month, meant to be paid out over the course of the plaintiff's lifetime. Unfortunately, this structure allowed predatory financial institutions to target low-income, often desperate victims, which subsequently acquired the settlement dollars in return for immediate, pennies on the dollar lump-sum payments to the victims. As a result, what was intended as a method of restorative justice became diluted, with only transient benefit for those experiencing a lifetime of harm. This elevates the importance of further lead abatement in affected communities, as it impossible to reverse the effects of exposure and efforts to ameliorate these effects are flawed at best.

Taking these factors into consideration, the bipartisan Infrastructure Investment and Jobs Act's plan to spend $15 billion in lead pipe removal is a wise investment and the next logical step in addressing the difficult challenge of ubiquitous lead exposure. As policymakers and public health officials continue to embrace the treatment of violence as a public health issue, this approach stands as a textbook example of a well-thought out public health intervention. Although some benefits, such as educational achievement, will be evident in the near-term, future public health officials, decades in the future, are likely to discover that this historic investment in lead pipe removal resulted in significant violence reduction for generations to come.

## Author Contributions

KF conceived of the manuscript. All authors contributed equally to the drafting, editing, and revisions of the manuscript.

## Funding

KF receives funding from the Health Alliance for Violence Intervention.

## Conflict of Interest

KF receives funding from the Health Alliance for Violence Intervention. The remaining authors declare that the research was conducted in the absence of any commercial or financial relationships that could be construed as a potential conflict of interest.

## Publisher's Note

All claims expressed in this article are solely those of the authors and do not necessarily represent those of their affiliated organizations, or those of the publisher, the editors and the reviewers. Any product that may be evaluated in this article, or claim that may be made by its manufacturer, is not guaranteed or endorsed by the publisher.
